# Investigating the emergence of sex differences in jealousy responses in a large community sample from an evolutionary perspective

**DOI:** 10.1038/s41598-021-85997-7

**Published:** 2021-03-22

**Authors:** Per Helge H. Larsen, Mons Bendixen, Trond Viggo Grøntvedt, Andrea M. Kessler, Leif Edward Ottesen Kennair

**Affiliations:** Department of Psychology, Norwegian University of Science and Technology, 7491 Trondheim, Norway

**Keywords:** Human behaviour, Sexual selection

## Abstract

Sex differences in jealousy responses to sexual and emotional infidelity are robust in samples of heterosexual adults, especially in more gender egalitarian nations. However, investigations of when and how these differences develop have been scant. We applied two forced choice infidelity scenarios in a large community sample of high school students (age 16–19, *N* = 1266). In line with previous findings on adults using the forced choice paradigm, adolescent males found the sexual aspect of imagined infidelity more distressing than adolescent females did. Nevertheless, there was no effect of age on the jealousy responses, and age did not moderate the sex difference. There were neither any effects of three covariates (having had first sexual intercourse, being in a committed romantic relationship, and sociosexuality), neither as markers of pubertal maturation nor as psychosocial environmental stimuli. Future research needs to investigate even younger samples in order to specify at what age the sex difference in jealousy responses emerges.

## Introduction

The main adaptive function of romantic jealousy is hypothesized to be retention of access to a valuable mate, and jealousy responses are therefore expected to be activated by relationship threats^1^. These threats involve partner sexual infidelity, defection from the relationship, and threats coming from potential mate poachers. Building on Trivers’^2^ parental investment theory, it is hypothesized that romantic jealousy evolved as a response to the recurrent adaptive problems of paternity uncertainty and potential investment loss^3^. Because sexual infidelity has potential significant higher fitness costs for men than for women, adaptations for identifying and preventing cuckoldry, including increased sensitivity and vigilance towards cues of sexual infidelity resulting in increased sexual jealousy, was more relevant for men^3^. If he is cuckolded, he may end up being an evolutionary dead end. Like females of all mammalian species women do not have parental uncertainty, however, unlike most mammalian species men invest in offspring. Women’s adaptive problem through evolutionary history has therefore primarily been to secure sufficient investment in the relationship and her offspring. If men divert investment from her to other women, this entails fitness costs for her. If he merely has sex with another partner, without diverting resources towards the new partner and potential offspring, this has limited costs for her. However, for how young age groups of men has the function of romantic jealousy been relevant throughout evolutionary history? This study investigates when this sex differentiation emerges in a large, community sample of adolescents.

Given the differences in costs for women and men entailed by different infidelity types, a sex difference in responses to types of infidelity is hypothesized. Men, relative to women, are expected to experience more jealousy in response to sexual infidelity, while women, relative to men, should experience more jealousy in response to emotional infidelity. This sex difference has been found using various methodologies^3–8^ and appears robust and replicable^9–12^, especially in Scandinavia, encompassing some of the world’s most gender egalitarian cultures^4,13–15^. Thus, while Tinbergen’s^16^ ultimate questions of function (prevent cuckoldry and infidelity) and phylogenetic history (as father investment and pair-bonding arose) have been addressed, and the proximate, psychological mechanisms described^1,17,18^, there has been little attention given to the ontogenetic development of jealousy. Recently romantic jealousy has been studied from a lifespan development perspective^19^, while the ontogenetic development and emergence of sex differences in adolescent romantic jealousy has not been addressed in detail. In this study, we therefore investigate at what age the sex difference in jealousy responses emerges in a large community sample of adolescents.

### The development of sex differences in jealousy

Previous developmental studies investigating topics related to jealousy have tended to focus on what might more aptly be classified as envy (e.g.^20^), and although the two might be similar colloquially, they are distinct emotions serving distinct functions^1^. The function of envy might best be described as motivating actions in order to obtain coveted benefits someone else has that one lacks oneself, whereas the function of jealousy is hypothesized to “motivate behavior designed to ward off threats to valued relationships with behavior ranging from vigilance to violence”^1^^, p. 156^. Several studies have investigated e.g. friendship envy in adolescents^21,22^ and sibling envy^23^.

Studies investigating jealousy in adolescence have tended to measure jealousy in a global, undifferentiated manner, without considering responses to different types of romantic threats^24^. Others have studied adolescents using the Friendship Jealousy Questionnaire^25^, or the Multidimensional jealousy scale^26^. These studies have examined phenomena related to romantic jealousy such as when romantic rivalry develops. Attractiveness-based social judgement biases toward same- and opposite-sex targets increased with targets’ age, in line with reproductive relevance^27^. In a sample of 7th, 9th, and 11th graders, both being jealous of a partner and being the subject of partner’s jealousy predicted likelihood of perpetrating teen dating violence^24^. No sex difference in jealousy was found among 13 and 16-year-old adolescents when their partner was around the opposite sex^28^. Further, relationship length was positively associated with increased jealousy responses during late adolescence, and females were more jealous than males although there was no specification of type of jealousy^25^. However, only two previous studies with adolescents^19,29^, have investigated romantic jealousy applying the most commonly used paradigm (Forced choice) developed by Buss and colleagues^3^. In a sample of undergraduate students (ages ranging from 15 to 25 years, with a mean age of 19 years), Shackelford et al.^29^ replicated the sex difference in distress to type of infidelity. The study focused on forgiveness or breakup following sexual or emotional infidelity. Relative to women, men found it harder to forgive and were more likely to break up the relationship following sexual infidelity compared to emotional infidelity by partner. Age did not affect this sex difference. The sample was relatively small (N = 256), and the age of the sample overlapped substantially with well-studied college and university samples. Similarly, a recent study by de Visser et al.^19^ investigated associations between responses to jealousy scenarios and a variety of individual differences (e.g. education, lifetime number of sexual partners, relationship status) in a population representative sample. The study also included a subsample of 16 to 19-year-olds which also showed the sex difference in jealousy response, but neither of the above studies explicitly investigated when the sex difference emerged, most likely due to small samples of individuals in early adolescence. Thus, how jealousy develops during adolescence remains largely unaddressed, and there is a need to investigate the ontogeny of romantic jealousy in younger samples.

Evolutionary developmental psychology seeks to test theoretically informative hypotheses, such as whether specific mechanisms depend on specific experience for their proper development^30^. An evolutionary developmental psychological perspective would not expect all evolved information-processing mechanisms to be present and expressed from birth, as different life stages posed different adaptive problems ancestrally^31^. Maintaining adaptively unnecessary systems for a specific age is biologically costly^32^, and selection should therefore have favored developmental programs that make the information-processing mechanisms come online just before the relevant life stage. This holds for physical adaptations such as teeth and female breasts, where the costs of being present from birth outweighs any benefits. This is also true for psychological mechanisms; for instance, the fear of heights does not come online before children starts moving independently^33^.

Natural selection designs adaptations that orchestrate developmental systems. Developmental systems that re-instantiate fitness-relevant designs are selected for across generations^31^. We can distinguish between different subtypes of adaptations. *Conditional* adaptations should come online given specific informational input from the external environment during ontogeny. In contrast, *deferred* adaptations’ developmental appearance should be expected to come online without depending heavily on experience, preparing organisms for stable features of ancestral environments^34^.

The problem of mate retention (avoiding mate poaching and partner infidelity) was a stable feature of reproductively mature humans’ species-typical environment, but it is conditional on reproductive maturity. The evolution of male sexual jealousy is contingent on reproductive pair-bonding and father investment. Cross-cultural evidence points to few men fathering children successfully before their third decade of life^35^. Therefore, it is unclear what function sexual jealousy would have had among very young males with low to no prospects of achieving or defending relationships. Because of their lower physical strength and social status, most of their mating opportunities would likely be very restricted, opportunistic and hazardous. Thus, we expect that sexual jealousy responses in men in long-term, reproductive relationships will come online prior to early twenties. For women, the emotional jealousy responses might be less specific as defecting friends, allies and romantic partners could plausibly be assessed with the same underlying, more domain-general mechanisms. These mechanisms are not as age specific as male sexual jealousy. Further, research into jealousy in sexual minorities suggests that only heterosexual males report increased distress to sexual infidelity^36^. Hence, it is primarily an increase in male sexual jealousy response that drives the sex difference. This is therefore the mental adaptation that needs to be described developmentally. As there is no other feedback mechanism than differential reproductive success to establish the effect of cuckoldry^37^, this is the most evolutionary relevant mechanism.

### Possible markers of developmental maturity and conditionally relevant experience

The literature shows inconsistent results for the role of experience on jealousy in adult samples^15,38^, with little influence of infidelity experience^39^. Overall, participant sex is the best predictor of infidelity reactions, and the sex differences in the forced choice distress response seem robust, and the sex difference is not subject to moderation, neither from personality^40^, nor interpersonal trust, beliefs about gender roles, nor belief in a culture of honor^41^. Investigations of the moderating role of attachment styles on sex typical responses to romantic jealousy has yielded inconsistent results; some studies find an influence of attachment style^42,43^, others find an influence only in men^44^, whereas others still find no influence^41^. However, Buss et al.^3^ hypothesized that “direct experience of the relevant context during development may be necessary for the activation of the sex-linked weighting of jealousy” (p. 255), but only found support for this hypothesis among adult men.

Own sexual and romantic experiences might be both indicators of pubertal maturation^45^ as well as input to developmental mechanisms. There are therefore different overlapping reasons to expect greater sex differentiated responses among adolescents that report having a romantic partner or having had their sexual debut. Earlier reproductive relevant experiences are linked to a faster life history^46,47^. Another indicator of faster life history is sociosexual orientation, a personality trait describing an individual’s preferences for casual sex and willingness to engage in sexual relations without commitment^48^. Individual differences in sociosexual orientation are present prior to high school age^49,50^. Previous studies expected sociosexual orientation to be related to sexual jealousy responses but found no relationship^4,41,51^. However, in an adolescent sample it may be that sociosexual orientation as a marker of earlier maturation may influence sex differentiated jealousy responses.

### The current study: aims and predictions

The objective of this study is to determine when the male increased sexual jealousy response comes online, and whether the sex difference in jealousy responses is the product of maturation or contingent upon specific domain relevant experiences. Given the dearth of research into the ontogenetic development and emergence of sex differences in romantic jealousy from an evolutionary perspective, we investigated a large community sample of adolescents, to see at what age the sex difference emerges, and whether the sex difference increases during the period from 16 to 19 years of age. Further, we will examine the influence of sexual experience, the participant’s current relationship status, and sociosexuality. Are reproductively relevant experiences necessary for proper activation and development of the distress response? As predicted by Buss et al.^3^ there might be some experiential activation following relationship experiences with high investment and commitment for males and females respectively.

Prediction 1: Given the existence of robust sex differences in romantic jealousy in Norwegian heterosexual student samples, we expect to find that this sex difference to emerge and widen during adolescence.

Prediction 2: We expect that both having had sexual intercourse and being in a committed relationship is associated with increased sex differentiation and sex typical jealousy responses during adolescence^3^.

Prediction 3: Despite the lack of influence of SOI in previous studies on adult participants^4,41,51^, SOI as a marker of faster life history (i.e. pubertal maturation) is expected to be associated with sex differentiated jealousy responses.

## Results

### Sex differences in experiences and sociosexuality

Chi-square analyses showed that a higher proportion of female (37.3%) than male (19.1%) students were currently in a committed relationship, *χ*^2^(1, *N* = 1259) = 49.10, *p* < 0.001, and more female (66.5%) than male (56.7%) participants reported having had their first sexual intercourse, *χ*^2^(1, *N* = 1206) = 12.18, *p* < 0.001. Independent samples t-tests showed that male students (*M* = 2.16, *SD* = 1.72) reported slightly more casual sex (SOI-Behavior) than female students (*M* = 1.93, *SD* = 1.18), *t*(1174) = 2.70, *p* = 0.007, *d* = 0.16. Further, male students (*M* = 6.29, *SD* = 2.25) reported markedly less restricted sociosexual attitudes than female students (*M* = 4.41, *SD* = 2.28), *t*(1198) = 14.13, *p* < 0.001, *d* = 0.83, and markedly higher levels of sociosexual desire: Males *M* = 4.38, *SD* = 2.27; Females: *M* = 2.57, *SD* = 1.67, *t*(1137) = 15.52, *p* < 0.001, *d* = 0.93.

### Simple sex differences in jealousy responses

**Figure 1:**
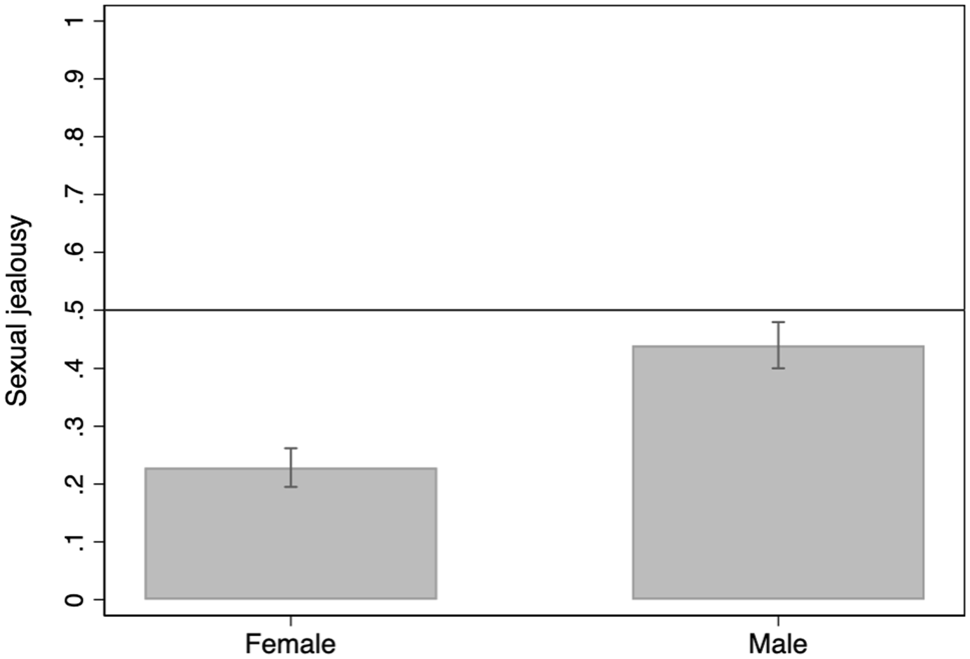
Proportion of female and male students (95% CIs) reporting being more upset by the sexual aspect of the infidelity on Scenario 1. Scores above solid line indicate being more upset by the sexual aspect of the infidelity scenario.

**Figure 2:**
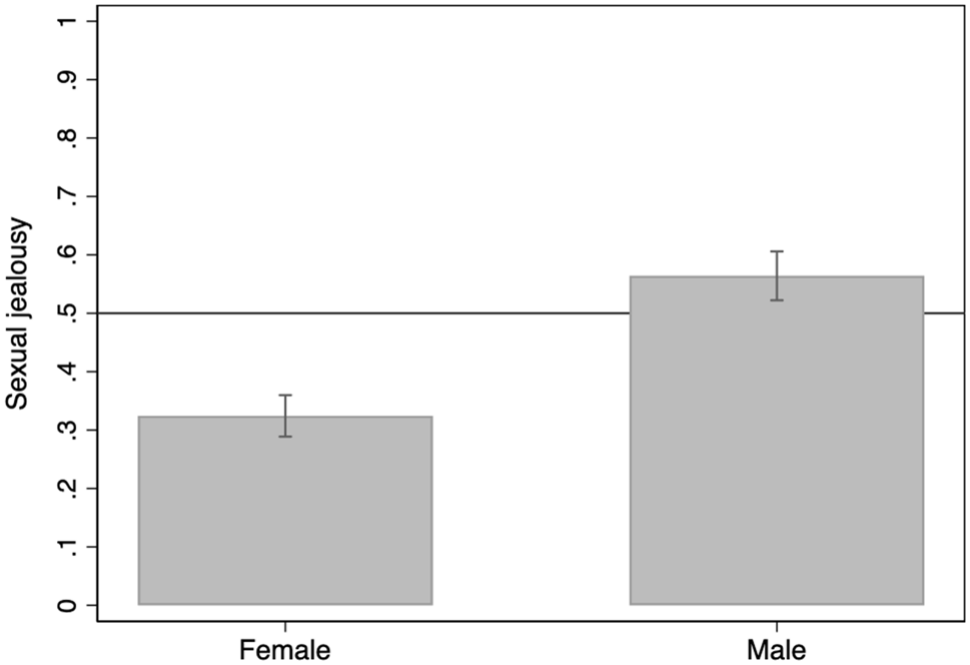
Proportion of female and male students (95% CIs) reporting being more upset by the sexual aspect of the infidelity on Scenario 2. Scores above solid line indicate being more upset by the sexual aspect of the infidelity scenario.

Relative to female students, male students reported being more jealous of the sexual aspect of the infidelity in both scenarios: Scenario 1, *χ*^2^(1, *N* = 1,216) = 61.03, *p* < 0.001, *r*_tau_ = 0.22. For Scenario 2: *χ*^2^(1, *N* = 1,210) = 69.31, *p* < 0.001, *r*_tau_ = 0.24. The proportion opting for the sexual aspect of infidelity for the two sexes is shown in Figs. 1 and 2.

### Multivariate analyses of sex differences in jealousy responses

**Figure 3:**
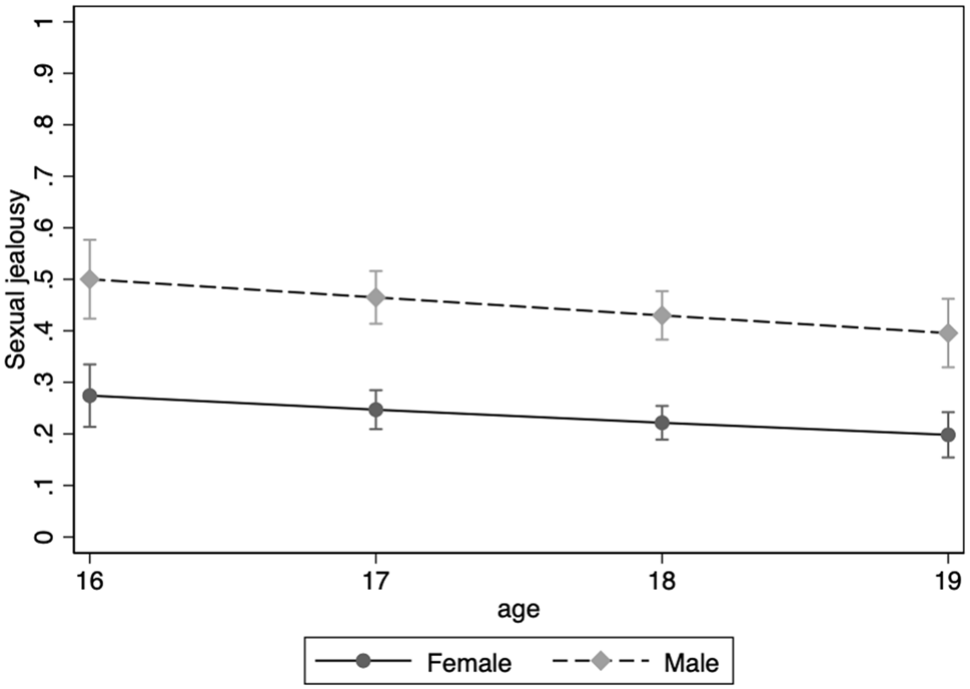
Proportion of female and male students reporting being more upset by the sexual aspect of infidelity at ages 16 through 19 on Scenario 1.

Next, we performed logistic regression analyses for the binary responses in Scenario 1 and Scenario 2. The analysis models the probability of a positive outcome given a set of regressors/predictors. We report on odds ratios (OR) and their 95% CI’s throughout. In Model 1, we added Age, Relationship status, and Sexual debut to the effect of Sex. Further, we added the three sociosexuality components in Model 2. In Scenario 1, Sex predicted jealousy response (OR = 2.65 [2.04, 3.43], *z* = 7.35, *p* < 0.001) with male students being more upset by the sexual aspect of infidelity than female students in Model 1. Neither Age (OR = 0.87 [0.75, 1.01], *z* = –1.83, *p* = 0.067), Relationship status (OR = 1.01 [0.73, 1.38], *z* = 0.05, *p* = 0.962), nor Sexual debut (OR = 1.05 [0.78, 1.41], *z* = 0.33, *p* = 0.739) had an effect on jealousy responses over and above the effect of Sex. Figure 3 shows the sexual jealousy scores for Scenario 1 across age for female and male students when the effects of Relationship status and Sexual debut were accounted for. Further, the effect of Sex was not moderated by age (*p* = 0.891), Relationship status (*p* = 0.131) or Sexual debut (*p* = 0.952). None of the separate tests of main and interaction effects for Age, Relationship status, and Sexual debut were significant.

Adding the three components of sociosexuality in Model 2 suggest that neither SOI-behavior (OR = 1.04 [0.94, 1.16], z = 0.78, *p* = 0.437), SOI-attitudes (OR = 1.06 [0.99, 1.13], z = 1.65, *p* = 0.100), nor SOI-desire (OR = 1.02 [0.94, 1.10], *z* = 0.38, *p* = 0.702) affected the jealousy responses. Also, neither component interacted with the effects of Sex in Model 2. The sex difference in sexual jealousy was not affected by level of unrestricted sociosexuality. The separate tests of main and interaction effects for SOI-behavior, SOI-attitudes, and SOI-desire showed that higher levels of unrestricted attitudes were significantly associated with jealousy responses (i.e., they were more upset by the sexual aspect) (*z* = 2.03, *p* = 0.043, *r* = 0.14), and that this effect was significantly moderated by sex (*z* = 2.00, *p* = 0.046). The association was somewhat stronger for males than for females. No other effects were significant.

**Figure 4:**
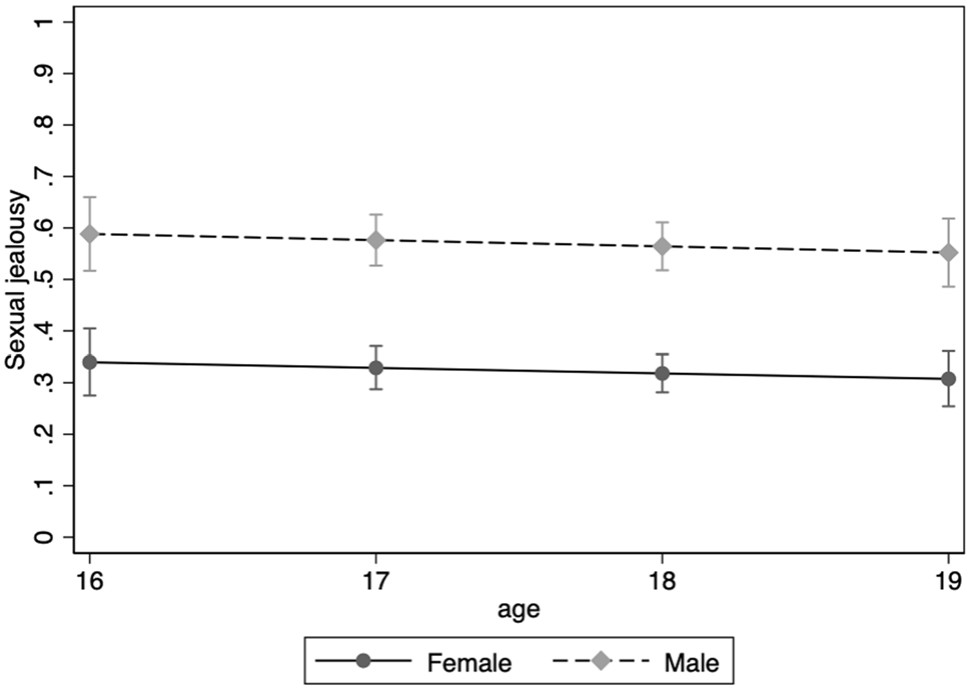
Proportion of female and male students reporting being more upset by the sexual aspect of infidelity at ages 16 through 19 on Scenario 2.

For Scenario 2, being male predicted being more upset by the sexual infidelity aspect (OR = 2.78 [2.17, 3.56], *z* = 8.08, *p* < 0.001). Neither Age (OR = 0.95 [0.84, 1.10], *z* = –0.68, *p* = 0.499), Relationship status (OR = 1.04 [0.76, 1.41], *z* = 0.29, *p* = 0.773) nor Sexual debut (OR = 1.11 [0.84, 1.47], *z* = 0.75, *p* = 0.451) affected the jealousy responses. Figure 4 shows the sexual jealousy scores for Scenario 2 across age for females and male students when the effects of relationship status and sexual debut were accounted for. The effect of Sex was not moderated by Age (*p* = 0.478), Relationship status (*p* = 0.803) or Sexual debut (*p* = 0.632). Similar to Scenario 1, none of the separate tests of main and interaction effects for Age, Relationship status, and Sexual debut were significant.

When the three components of sociosexuality were added in Model 2, neither SOI-behavior (OR = 1.00 [0.90, 1.11], *z* = 0.04, *p* = 0.964), SOI-attitudes (OR = 1.06 [0.99, 1.13], *z* = 1.64, *p* = 0.102), nor SOI-desire (OR = 1.08 [1.00, 1.16], *z* = 1.88, *p* = 0.059) significantly affected the jealousy responses. However, both SOI-attitudes (*z* = 2.54, *p* = 0.011) and SOI-desire (*z* = 2.65, *p* = 0.008) significantly predicted jealousy responses when entered separately in Model 2. The separate tests of main and interaction effects for SOI-behavior, SOI-attitudes, and SOI-desire showed that higher levels of unrestricted attitudes (*z* = 2.42, *p* = 0.016, *r* = 0.15) and desire (*z* = 2.17, *p* = 0.030, *r* = 0.16), were significantly associated with jealousy responses (i.e., more upset by the sexual aspect). These main effects were not significantly moderated by sex. None of the effects for SOI-behavior were significant.

## Discussion

In a large community sample of adolescents, we found sex differentiation in jealousy responses to infidelity scenarios at all ages from 16 to 19. The jealousy responses were neither affected by sexual or romantic experiences nor by respondent sociosexual orientation. Prior to this study, it was known that in adult heterosexual samples there is a sex difference in responses to jealousy scenarios using a forced choice paradigm^1^. This sex difference has proven especially robust in Scandinavian young adult samples^4,14,15^. Previously only Shackelford et al.^29^ and de Visser et al.^19^ have considered the sex difference in romantic jealousy among adolescents. However, Shackelford et al.^29^ only included a small number of participants younger than 20 and de Visser et al.^19^ focused primarily on life-span development. None of these considered the ontogenetic development and emergence of the sex difference in romantic jealousy during adolescence specifically and in detail. The current study therefore set out to discover when these sex differences emerge in a large community adolescent sample. Further, we considered how relationship status, sexual debut and sociosexuality might moderate the development of the sex difference in romantic jealousy – indicators of both psychological and pubertal maturation or life history^52^ and relevant experiences^3^.

Although our sample represents the largest group of young adolescents yet studied, we were unable to detect at what age the sex difference comes online. There was no interaction with age. Further, despite what evolutionary theories predict^3^, there was neither any effect of relationship status, sexual status, nor sociosexuality on the development of sex differentiation of jealousy responses. None of our predictions were supported. The sex difference appears to develop earlier than age 16, and it is fully established at that age.

Suggesting that these results were obvious a priori is not convincing. The analyses of de Visser et al.^19^ and Shackelford et al.^29^ do not really allow for any extrapolation to the age group considered here, due to low number of participants in the relevant age and no specific attention to processes in the ages 16 to 19. The current data were also collected prior to de Visser et al.^19^. Nevertheless, an evolutionary perspective suggests that adaptations develop or come online strategically, just prior to being adaptive relevant^32^.

This leaves us with some evolutionary developmental conundrums. The increased male heterosexual sexual jealousy response seems to be present at an early age and not contingent upon the specific domain relevant experiences we measured in this study. What function this specific emotional response to sexual infidelity might have played in young males in an evolutionary context is therefore unclear. None of the current evolutionary psychology approaches has specified any function for increased male sexual jealousy in adolescents too young to be troubled with cuckoldry, which is the main function of male, heterosexual sexual jealousy. The acquisition of committed, long-term partners that young men were able to defend from older, more formidable rivals with higher status was most likely evolutionary rare^35^. Further, at lower ages, it is unclear what, if any function early adolescent males’ increased focus on physical aspects of their romantic partners’ infidelity should serve. It is possible that the developmental appearance of sexual jealousy simply prepares the individual for later life, serving no function during adolescence. However, the current findings warrant further theoretical development. If anything, increased emotionality in young males might put them in harm’s way from older, more formidable men, rather than improving any relevant paternal certainty. Nevertheless, at some point when moving towards younger ages, the responses the participants would be reporting on might not be the functional jealousy of adults, but some form of a proto-sexual jealousy, with other possible specific or deferred functions. This might also be reflected in “puppy love”^53^ or other roleplay type of love bonds between children.

Finally, our community sample evinced adequate variance in the different sexual and relationship experiences to detect any conditional developmental effects in jealousy responses. At this point we would need further studies of early puberty to address the question of the emergence and development of this sex difference. Based on these results, our preliminary conclusion must be that increased heterosexual male sexual jealousy matures primarily as part of puberty, independent of specific experiences. However, the function of this response at this age remains unclear, as it would seem it serves no anti-cuckolding function, while retaining, especially in concert with Young Male Syndrome tendencies^54^, the cost of violent conflict with more formidable older males.

### Limitations and future research

While this is the largest, youngest adolescent sample to be studied with the forced choice paradigm, we were unable to discover the point in development when the sex difference emerges. Future studies therefore need to investigate jealousy responses in even younger samples. However, doing so would introduce ethical and methodological challenges. As we consider continually lower ages, the scenarios will at some age stop being meaningful, or developmentally relevant to the participants. We need to consider the possibility that the males’ increased proto-sexual jealousy develops early in puberty. Future studies should possibly also go beyond self-report and investigate specific design features of jealousy and their development in men and women using longitudinal designs, including other developmentally relevant factors. Such a design might better address causality than our cross-sectional design. However, in this first in-depth of the emergence of the sex difference of jealousy responses, our focus was not primarily on causes, but on at what age this difference appears. Also, the community sample consisted of four representative age cohorts^55^, and therefore ought to be adequate to address age trends. Further, the current approach to the development of jealousy is limited to test a specific set of predictions and does not address several other socio-cultural and intrapsychic factors, that may be of interest in future studies.

Conclusions from a Norwegian sample are admittedly culturally limited, to some degree^56^—and in any case subject to evolutionary mismatch^57^. Known cultural and socioecological variation that contribute to variation in jealous responses include more parental investment and less extramarital sex, which predict more severe jealousy responses across cultures^12^. Despite the importance of cultural and socioecological factors, similar forced-choice patterns have been found in cultures as diverse as China^40^, Korea^58^, Japan^12,58^, Sweden^14^, Norway^4^, England, Romania^59^, Germany, Netherlands^60^, Spain^61^, India, and among the Hadza, Himba, Karo Batak, Mayangna, Mosuo, and the Shuar^12^.

Finally, the current study suffers from the same limitations that hold true for all hypothetical scenarios, and the forced choice paradigm. Continuous measures and experienced jealousy may also be studied in the future in this age group.

## Conclusions

We conclude that the sex difference in sexual versus emotional jealousy develops earlier than the age of 16. There was no widening of the sex difference as a function of age in this largest and youngest sample of adolescents to consider sex differences in jealousy. This was surprising, given that few ancestral males of this age are thought to have established long-term reproductive relationships, reproduced with high likelihood, or would have been in a position to defend their partners against older, more formidable rivals with higher status. Future research needs to develop methodology to consider younger age groups. However, one must also consider ethical challenges in asking younger samples questions about sexual or physical infidelity. Further, maturational, or experiential indicators, such as sexual debut and relational status, neither influenced the sex difference. Studies taking these considerations into account are needed in order to uncover when and how the green-eyed monster awakens.

## Methods

### Design and subjects

A cross-sectional, community sample study on Sexual Health and Sexual Harassment was carried out in Central Norway in the time period from May 2013 to May 2014. Students enrolled in 17 high schools were invited to respond to a web-based questionnaire. Responses were carefully screened for highly inconsistent, monotonous, and unserious responding. After removal of students reporting non-heterosexual orientation, 1,266 high-school students aged between 16 and 19 years old (726 female, *M* = 17.7, *SD* = 0.87; 540 male, *M* = 17.6, *SD* = 0.81) were eligible for analyses. Further information on the sample characteristics, representativeness, and design are found elsewhere^49,55^.

### Procedure

Prior to data collection we provided written information about the study content and main purpose of the project to students, their parents and the school staff. The school administered the informed consent form. To get access to the electronic questionnaire (a login code), each student needed to return the consent form to the school. All participants were above 16 years of age and could therefore consent to the study by signing the informed consent forms, following Norwegian ethical regulations. Students could respond to the questionnaire at home or in the classroom. To ensure anonymity and confidentiality the school made arrangements for group administration. Because the questionnaire covered sensitive and personal topics on health and sexuality the school's public health nurses were available for consultation during the weeks that the survey took place. The Regional Committee for Medical and Health Research Ethics, Regional Etisk Komité: Midt (English: REC Central), approved the procedure (REK: 2013/408).

### Measurements included in the current study

#### Relationship status

The participants reported whether they were in a committed relationship and if so, the duration of the relationship. For those who reported being in a committed relationship for three months or more, relationship status was coded 1 = Yes /0 = No.

#### Sexual experience

The sexual intercourse status of those who reported having had sexual intercourse was coded 1 = Yes /0 = No.

#### Sociosexuality

Participants filled out the revised Sociosexual Orientation Inventory (SOI-R, 62). The 9-item measure reflects three intercorrelated components: behavior, attitudes, and desire. Each of the three components had excellent internal consistency: SOI-Behavior (*α* = 0.90), SOI-Attitudes (*α* = 0.88), and SOI-Desire (*α* = 0.89). Standard scaling and scoring was applied^62^. Scales’ scores ranged from 1 to 9, and higher scores reflect less restricted sociosexuality.

#### Infidelity scenarios

In the first scenario we asked participants to imagine being in a committed relationship where their partner has developed an interest for an extra-pair individual, and then asked whether participants would be most distressed by their partner developing an (A) emotional (but not sexual) relationship, or (B) sexual (but not emotional) relationship. The second scenario asked the participant to imagine their partner developing both an emotional and a sexual relationship, and choose which aspect would be most distressing, the fact that their partner is (A) having sex with someone else, or (B) developing emotional bonds to someone else. These scenarios are the same as two of the scenarios used by Buss et al.^58^. For both scenarios, a jealousy score of 1 reflects that the sexual infidelity was more distressing than the emotional infidelity. A score of 0 reflects that the emotional aspect was more distressing. The scores for the two scenarios correlated moderately (*r*_tau_ = 0.43).

### Statistics

The data were analyzed using Stata/MP 16.1 for Mac.

### Ethical statement

APA standards and guidelines were followed in the conduct of the study.
